# Breast cancer knowledge and screening practices among undergraduates in a Nigerian tertiary institution, Southwest Region

**DOI:** 10.4314/ahs.v22i4.4

**Published:** 2022-12

**Authors:** Abayomi Yusuf, Ifeoma Okafor, Tope Olubodun, Olanrewaju Onigbogi

**Affiliations:** 1 Department of Community Health & Primary Care, College of Medicine, Idi-Araba 100254 Lagos, Nigeria; 2 Department of Community Health, Lagos University Teaching Hospital, Idi-Araba 100254 Lagos, Nigeria

**Keywords:** Knowledge, practice, breast cancer, screening, young people, Nigeria

## Abstract

**Introduction:**

Breast cancer is the most diagnosed female malignancy in Nigeria. This study aimed to assess the knowledge and practice of breast cancer screening among female undergraduates in a tertiary institution in Southwest Nigeria.

**Methods:**

This cross-sectional study recruited 350 female undergraduates of a public university in southwest Nigeria using a multi-stage sampling method involving simple random sampling. A structured self-administered questionnaire was used for data collection. Epi info 7 was used for data analysis, level of significance was set at 5% (p<0.05).

**Results:**

The majority, 316(90.3%) had poor knowledge of breast cancer and screening and 340(97.1%) had positive attitude towards screening. Only 185(52.9%) had done breast self-examination and 16(4.6%) had done clinical breast examination. The student's year of study was significantly associated with knowledge of breast cancer (p = 0.002) Marital status (p=0.039) and attitude towards breast cancer screening (p<0.001) were significantly associated with breast self-examination. Students in their third year were 6 (2 – 16) times more likely to have good knowledge of breast cancer (Adjusted Odds Ratio 5.87, Confidence Interval 2.17 – 15.86).

**Conclusion:**

Overall knowledge and practice of breast cancer screening were poor, but students' attitude towards screening was positive. Health education on breast cancer and screening is recommended.

## Introduction

Breast cancer is one of the commonest malignancies that affect women globally impacting over 2.1 million women each year.^1^ It is the most diagnosed cancer in Nigeria and has a significant public heath burden because of the associated morbidity and mortality. 2 There has been a steady increase in the incidence of breast cancer in Nigerian women from 13.7 per 100,000 in the period 1960 -1969 to 24.7 per 100,000 in 1998 - 1999 to 38.7 per 100,000 in 2008 to 54.3 per 100,000 in 2010.^2^,^3^ In Nigeria, survival rate is very low as most women present at advanced stages of the disease.^3^

Late presentations of breast cancer have been associated with poor knowledge of its symptoms.^4^,^5^ Other factors contributing to late presentations include self-denial, superstition, fear of mastectomy amongst others.^4^ Early detection remains a major effective approach that should be employed in combating the disease and this is only possible with the aid of organized screening practices such as breast self-examination (BSE), clinical breast examination (CBE), mammography and screening magnetic resonance imaging (SMRI) as well as efficient treatment modalities.^5^,^6^ Breast cancer screening aims to detect early disease with resultant improved prognosis and quality of life from less radical treatments.^6^ An ideal screening test is characterized by simplicity, effectiveness and affordability. 5 Breast cancer screening modality provided depends on a number of factors like age and family history. Although these screening practices are not readily accessible, BSE is cheap and easily taught to women.^6^

Women should be made to understand the benefits of detecting the disease early which include breast tissue conservation, overall good prognosis, the possibility of cure and total recovery from the condition. This knowledge has the potential of encouraging women to examine their breasts regularly and present early to a health facility if they notice any sign or feel any symptom. This study assessed the knowledge, attitude and practice of breast cancer screening among female undergraduates of a Federal Tertiary Institution in Abeokuta, Southwest Nigeria. The results are expected to provide critical information for the design of effective breast cancer prevention campaign programmes among young people in the country and develop strategies for accessible means of detecting the disease in its early stages. The study was also expected to contribute to the existing body of knowledge about breast cancer screening among young females in academic communities.

## Methods

### Study design, setting and selection of participants

This cross-sectional study was carried out at the Federal University of Agriculture, Abeokuta, Ogun State (FUNAAB) which comprises ten (10) colleges. The participants were female students of the university. Only those who were registered full-time in first year to final year and between the ages of 16 to 35 years were included while postgraduate and part time students were excluded.

The minimum sample size was determined using Cochran formula where prevalence p, represents the proportion (71.6%) of women who had good knowledge of breast cancer screening in a study aimed at evaluating the awareness and practices of breast cancer screening on early detection of breast cancer.^6^ The level of accuracy was set at 5%. The calculated minimum sample size was 313 with an additional 10% of this number added to make up for non-responses. The final sample size used for the study was therefore rounded up to 350.

A multistage sampling method was used to select participants in this study.^7^ In stage one, simple random sampling by balloting was used to select seven out of ten colleges in the institution that were to participate in the study. In stage two, simple random sampling (balloting) was used to select one department each from the seven colleges. In stage three, simple random sampling, by balloting was used to select two levels from each of the seven selected departments. In stage four, simple random sampling (balloting) was used to select twenty-five students from each of the selected departments.

### Data sources and measurement

Data was collected in September 2018 using a structured, self-administered questionnaire adapted from similar study conducted in Southeast Nigeria.^4^ The questionnaire was pretested at another tertiary institution in Abeokuta, Ogun State. The questionnaire elicited respondent's socio-demographic characteristics such as age, marital status, year of study among others. Knowledge of the respondents on breast cancer was assessed by asking questions on risk factors for breast cancer, symptoms, treatment options and benefits of early detection. Next was knowledge of breast cancer screening methods which covered breast self examination (age of onset, frequency, and timing), clinical breast examination, breast ultrasound scan and mammography. The attitude of the respondents to breast cancer screening was assessed by indicating their agreement or disagreement to a set of statements. Finally, information on the respondents' practices regarding breast cancer screening were elicited.

The questionnaires were administered and retrieved over two days by the lead researcher and six (6) research assistants (medical undergraduates) who were recruited and trained for a day on methods of data collection to ensure standardization in the administration and retrieval of the questionnaires. Data were collected from students in their lecture halls before their lectures or during their break periods. Each respondent spent about 15 to 20 minutes to fill the questionnaire and any problem encountered by them were appropriately attended to by the lead researcher and research assistants. The completed questionnaires were retrieved immediately from a designated point within the lecture halls.

### Statistical analysis

Data was entered and analysed using Epi info 7 Software and summarized with proportions, mean, and standard deviation. To test association between categorical variables, bivariate analysis was done using Chi-square test and p value was set at 5% (0.05) significant level. Multivariable logistic regressions were done to eliminate confounders and determine predictors of good knowledge and screening practice.

The knowledge of respondents on breast cancer was graded based on their knowledge of breast cancer, risk factors, symptoms, treatment options and benefits of early detection of breast cancer. Each correct response was scored one (1) point, otherwise zero (0). The total score ranged from 0–27 points and converted to percentage. Scores were computed as ratio of respondent total score to maximum score obtainable. Knowledge was graded as poor if less than 50% and graded as good if greater than or equal to 50%.

The knowledge of respondents on breast cancer screening was graded based on their knowledge of breast cancer screening methods (breast self-examination, clinical breast examination breast ultrasound scan and mammography). Each correct response was scored one (1) point, otherwise zero (0). The total score ranged from 0–20 points and converted to percentage. Scores were computed as ratio of respondent total score to maximum score obtainable. Knowledge of breast cancer screening methods was graded as poor if respondent scores less than 50% and good if greater than or equal to 50%.

Fifteen (15) Likert statements were used to assess respondents' attitude towards breast cancer screening based on five-point scale responses - Strongly Agree, Agree, Disagree, Strongly Disagree and Indifferent. The highest score was 5 (i.e., for Strongly Disagree) and the lowest score was 1 (i.e., for Strongly Agree). The highest score was 75 while the lowest score was 15. The mid-point between the maximum score and the minimum score was 45. Scores greater than or equal to 45 were graded positive attitude and below, negative attitude. Respondents' screening practices were assessed with questions on whether they had done breast self examination, clinical breast examination and breast scan. Use of mammography was not assessed due to age considerations. It is usually recommended from 40 years of age but only students aged 16 to 35 years were included in the study. Practice was not scored.

### Ethical considerations

Ethical approval was obtained from Health Research Ethics Committee of Lagos University Teaching Hospital (LUTH) (Health Research Committee Assigned Number: ADM/DCST/HREC/APP/370). A written informed consent was obtained from respondents before enrolment into the study. They were informed on the scope, objective of the study and for confidentiality. All questionnaires were anonymous, and participation was voluntary.

## Results

### Socio demographic characteristics

The mean age of the respondents was 20.63 years (SD ± 2.488 years), 304(86.9%) of respondents were of the Yoruba tribe, 341(97.4%) were classified as single/never married, and 280(80.0%) were of the Christian faith. Ninety-eight (28.0%) of the respondents were in their third year of training, 327(93.4%) were unemployed and the mean monthly income/allowance was 17, 562.86 ± 27, 861.62 Naira (approximately 48.12 ± 76.33 USD at 365 Naira to 1 USD) (Table 1).

**Table 1:** Socio-demographic characteristics of the respondents

Variable	Frequency (n=350)	Percentage (%)
**Age (years)**		
16 – 20	174	49.7
21 – 35	176	50.3
Mean ± SD = 20.63 ± 2.488		
**Ethnic group**		
Hausa	1	0.3
Igbo	36	10.3
Yoruba	304	86.9
Others	9	2.7
**Religion**		
Christianity	280	80.0
Islam	70	20.0
**Marital status**		
Married/co-habiting	9	2.6
Single/never married	341	97.4
**Year of training**		
1^st^ – 2^nd^	128	36.6
3^rd^	98	28.0
4^th^ – 6^th^	124	35.4
**Employment status**		
Employed	23	6.6
Unemployed	327	93.4
**Monthly income/allowance**		
<10,000	85	24.3
10,000 – 14,000	124	35.4
≥15,000	141	40.3
Mean ± SD = 17562.86 ± 27861.617		
**Family history of breast cancer**		
No	322	92.0
Yes	28	8.0

### Knowledge of breast cancer

Majority, 341(97.4%) of the respondents were aware of breast cancer. Media (television, radio and internet) was the major source of information, 260(76.2%) followed by books, 125(36.7). Only 28(8.0%) had previous family history of the disease.Of the respondents that had ever heard of breast cancer, only 40(11.7%) knew early menarche and late menopause 23(6.7%) as risk factors for breast cancer. However, 78(22.9%) of the respondents knew smoking as a risk factor. (Table 2).

**Table 2:** Knowledge of breast cancer

Variable	Frequency (n=350)	Percentage (%)
**Ever heard of breast cancer (n = 350)**	341	97.4
*Source of information (n = 341)		
Books	125	36.7
Lecture	105	30.8
Media	260	76.2
Hospital	92	21.7
Conference/Seminar	74	21.7
Friends	106	31.1
Others	3	0.9
*Risk factor of breast cancer (n = 341)		
Old age	41	12.0
Smoking	78	22.9
Alcohol	56	16.4
Early menarche	40	11.7
Late menopause	23	6.7
Not breastfeeding	61	17.8
Obesity	35	10.3
Estrogen oral contraceptives	82	24.0
I don't know	141	41.3
Others	8	2.3
**Presence of lump in the breast may indicate breast** **cancer (n = 341)**	161	47.2
*Symptom of breast cancer (n = 341)		
Breast mass	102	29.9
Swelling under armpit	66	19.4
Pain in the breast	213	62.5
Nipple discharge	133	39.0
Weight loss	50	14.7
Skin changes on the breast	97	28.4
Rashes or sores around the nipple	112	32.8
*Treatment option (n = 341)		
Surgery	184	54.0
Radiotherapy	77	22.6
Chemotherapy	158	46.3
Hormone therapy	23	6.7
Biological therapy	6	1.8
I don't know	59	17.3
*Benefit of early detection of breast cancer **(n = 341)**		
Reduces risk of death	210	61.6
Early treatment	248	72.7
Improves quality of life	91	26.7
Reduces cost of treatment	75	22.0
Improves outcome of disease	47	13.8
I don't know	24	6.0

Some 161(47.2%) of the respondents knew pain in the breast as a symptom of breast cancer. Only 184(54.0%) knew surgery as a treatment option, 158(46.3%) knew chemotherapy while 6(1.8%) knew biological therapy. Some 24(6.0%) of the respondents did not know the benefits of early detection of breast cancer. Overall, majority 316(90.3%) of the respondents had poor knowledge of breast cancer (Table 2).

### Awareness and knowledge of breast cancer screening methods

The majority 294(86.2%) of the respondents knew breast self-examination as a method of breast cancer screening, 191(56.0%) knew clinical breast examination, 169(49.6%) knew breast ultrasound scan and 114(33.4%) knew mammography. One in 10 of the respondents did not know about breast cancer screening methods. Overall, 270(77.14) of the respondents had poor knowledge of breast cancer screening (Table 3).

**Table 3:** Respondents' knowledge of breast cancer screening

Variable	Frequency	Percentage
*Breast cancer screening method known (n = 341)		
BSE	294	86.2
CBE	191	56.0
Breast ultrasound scan	169	49.6
Mammography	114	33.4
I don't know	35	10.3
Ideal age to begin BSE is 20 years (n = 294)	32	10.9
Ideal time interval for performing BSE is monthly (n = 294)	106	36.1
Best time to perform BSE is a week after period (n = 294)	111	37.8
BSE is done by the individual (n = 294)	264	89.8
Method of performing BSE (n = 294)		
Feeling the armpit with the hand	4	1.4
Feeling the breast with the hand	215	73.1
Inspecting the breast in front of the mirror	67	22.8
CBE is done by trained health workers (n = 191)	174	91.1
CBE is done with hand (n = 191)	44	23.0
Ideal time interval for performing CBE is yearly (n = 191)	22	11.5
Breast scan is done using ultrasound (n = 169)	69	40.8
Ideal age to begin mammography is 40 years (n = 114)	24	21.1
Time interval for performing mammography is yearly (n = 114)	20	17.5
**Overall knowledge of Breast cancer and screening**		
Poor knowledge	316	90.3
Good knowledge	34	9.7

### Attitude towards breast cancer screening

Attitude towards breast cancer screening was generally positive among the respondents 340(97.1%). Over half 187(53.4%) of the respondents strongly disagreed to the statement: “it is not necessary to advice all adult females to practice breast cancer screening”. About half 176(50.3%) of the respondents strongly disagreed to the statement: “it is immoral to touch one's breast”. Also, 132(37.7%) of the respondents strongly disagreed to the statement: “I am too young to have breast cancer” (Table 4).

**Table 4:** Respondents attitude towards breast cancer

Variable (n=350)	SD	D	I	A	SA
It is not necessary to advice all adult females to practice breast cancer screening	187(53.4)	81(23.1)	9(2.6)	27(7.7)	46(13.1)
I consider breast cancer examination embarrassing	168(48.0)	135(38.6)	18(5.1)	24(6.9)	5(1.4)
It is better one does not know they have cancer	236(67.4)	76(21.7)	20(5.7)	9(2.6)	9(2.6)
I can never have breast cancer	50(14.3)	64(18.3)	71(20.3)	43(12.3)	122(34.9)
I do not believe in the efficacy of the screening methods	124(35.4)	148(42.3)	49(14.0)	20(5.7)	9(2.6)
Breast screening is not culturally acceptable	115(32.9)	114(32.6)	74(21.1)	35(10.0)	12(3.4)
I will not do breast cancer screening because of my religious beliefs	208(59.4)	117(33.4)	19(5.4)	4(1.1)	2(0.6)
If I find something unusual in my breast, I will not need to consult a health worker but will only pray about it	210(60.0)	102(29.1)	17(4.9)	8(2.3)	13(3.7)
It is immoral to touch one's breast	176(50.3)	100(28.6)	42(12.0)	14(4.0)	18(5.1)
I am too young to have cancer	132(37.7)	106(30.3)	52(14.9)	23(6.6)	37(10.6)
I consider traditional medicine to be better than modern medicine in the treatment of breast cancer	160(45.7)	104(29.7)	61(17.4)	16(4.6)	9(2.6)
Diagnosis of breast cancer is a death sentence	193(55.1)	118(33.7)	26(7.4)	8(2.3)	5(1.4)
Breast cancer is not a serious disease	205(58.6)	107(30.6)	27(7.7)	7(2.0)	4(1.1)
Women with breast cancer should not be supported but pitied	259(74.0)	67(19.1)	10(2.9)	7(2.0)	7(2.0)
CBE should be performed only by a female physician	74(21.1)	77(22.0)	65(18.6)	68(19.4)	66(18.9)
**Attitude towards Breast cancer screening**					
Negative attitude	10(2.9)				
Positive attitude	340(97.1)				

SD- Strongly Disagree, D- Disagree, I- Indifferent, A- Agree, SA- Strongly Agree

### Breast cancer screening practices

Only about half, 182(52.0%) of the respondents were taught how to do breast self-examination and about a quarter were taught by their parents, 45(24.7%). Just about half, 185(52.9%) of the respondents had done breast self-examination. The major reason for not doing breast self-examination was that they did not know how to do it, 72(43.6%). Also, 25(13.5%) of the respondents had discovered an abnormality in their breast while performing breast self-examination (Table 5).

**Table 5:** Practice of breast cancer screening

Variable	Frequency (n=350)	Percentage (%)
**Taught how to do BSE (n = 350)**	182	52.00
**Taught by (n = 182)**		
Doctor	18	9.9
Friend	35	19.2
Nurse	38	20.9
Parent	45	24.7
Teacher	25	13.7
Others	21	11.5
**Done BSE**	185	52.9
**Does BSE monthly (n = 185)**	30	16.2
**Reason for not doing BSE (n = 165)**		
I am afraid I may have breast cancer	1	0.6
I don't get enough privacy	1	0.6
I don't have any symptom(s)	35	21.2
I don't know how to do BSE	72	43.6
I am afraid I may have breast cancer	1	0.6
I have not heard of BSE	33	20.0
I usually forget	19	11.5
It is immoral to touch one's breast	3	1.8
My culture and religion do not support it	1	0.6
I have not heard of BSE	33	20.0
**Discovered abnormality during practice of BSE for only** **respondents who had ever done BSE (n = 185)**	25	13.5
*Action taken after discovery of abnormality (n = 25)		
Prayed over it	3	12.0
Did some lab. Tests	3	12.0
Did nothing	10	40.0
Consulted a health worker	12	40.0
**Has undergone CBE (n = 350)**	16	4.57
**Time of last CBE (n = 16)**		
More than a year ago	11	68.8
Within the past one year	5	31.2
**Reasons for not practicing CBE (n = 334)**		
Consulting a doctor is expensive	116	34.7
I don't want a health worker touching my breast	18	5.4
I have not heard of CBE	125	37.4
It is embarrassing	10	3.0
Others	65	19.5
**Has done breast ultrasound scan (n = 350)**	9	2.57
**Has not done mammography (n = 350)**	350	100.0

Only, 16(4.6%) of the respondents had done clinical breast examination (CBE) and the reason for not doing clinical breast examination by most of the respondents was that they had not heard of clinical breast examination, 125(37.4%). Use of breast scan among the respondents was 9(2.6%) (Table 5).

### Factors associated with knowledge of breast cancer

There was a statistically significant association between respondent's year of study and knowledge of breast cancer (p = 0.002). Respondents in their third year of training had significantly better knowledge than those in other levels (Table 6). Following a multivariate logistic regression analysis using first and second years of training as reference, third year students were 6 (2 – 16) times more likely to have good knowledge of breast cancer (AOR 5.87, CI = 2.17 – 15.86) (Table 8).

**Table 6:** Factors associated with knowledge of breast cancer

	Knowledge of breast cancer				
Variables	Poor (%)	Good (%)	Total (%)	X^2^	P- value
	316(90.3)	34(9.7)	350(100.0)		
**Age (years)**					
16 – 20	153(87.9)	21(12.1)	174(100.0)	2.187	0.139
21 – 35	163(92.6)	13(7.4)	176(100.0)		
**Marital Status**					
Single/ never married	308(90.3)	33(9.7)	341(100.0)	0.021	0.606#
Married/co-habiting	8(88.9)	1(11.1)	9(100.0)		
**Year of training**					
1^st^ – 2^nd^	122(95.3)	6(4.7)	128(100.0)	12.439	**0.002**
3^rd^	80(81.6)	18(18.4)	98(100.0)		
4^th^ – 6^th^	114(91.9)	10(8.1)	124(100.0)		
**Member of family diagnosed with** **breast cancer**					
No	292(90.7)	30(9.3)	322(100.0)	0.725	0.334#
Yes	24(85.7)	4(14.3)	28(100.0)		

# Fisher's exact p-value

X^2^ Chi square test

**Table 8:** Predictors of good knowledge and practice of breast cancer screening

Good knowledge of breast cancer				
Variable	AOR	Lower CI	Upper CI	P- value
**Year** **of training**				
1^st^ – 2^nd^	1			
3^rd^	5.87	2.17	15.86	<0.001
4^th^ – 6^th^	2.85	0.93	8.79	0.068
**Practice of BSE**				
**Age (years)**				
16 – 20	1			
21 – 35	0.99	0.62	1.62	0.995
**Marital status**				
Single/Never married	1			
Married/co-habiting	0.15	0.02	1.21	0.075
**Year** **of training**				
1^st^ – 2^nd^	1			
3^rd^	0.93	0.54	1.62	0.808
4^th^ – 6^th^	1.24	0.70	2.20	0.461
**Practice of CBE**				
**Age (years)**				
16 – 20	1			
21 – 35	0.66	0.41	4.10	0.657
**Year** **of training**				
1^st^ – 2^nd^	1			
3^rd^	0.44	0.11	1.78	0.248
4^th^ – 6^th^	0.55	0.15	2.01	0.364

AOR: Adjusted Odds Ratio CI: Confidence Interval

### Factors associated with breast cancer screening practice

There was a statistically significant association between respondent's marital status and practice of BSE (p = 0.039). Respondents who were married/co-habiting had significantly better practice of BSE than those that were single/never married. Also, there was statistically significant association between attitude towards breast cancer screening and practice of BSE (p < 0.001) Respondents who had negative attitude towards screening has significantly poorer practice of BSE. (Table 7). Age, year of study, marital status, attitude and family history of breast cancer were not predictors of screening practice (Table 8).

**Table 7:** Factors associated with breast cancer screening

	Practice of BSE				
Variable	No (%)	Yes (%)	Total (%)	X^2^	P- value
	165(47.1)	185(52.9)	100(100.0)		
**Age(years)**					
16–25	84(48.3)	90(51.7)	174(100.0)	1.78	0.673
26–35	81(46.0)	95(54.0)	176(100.0)		
**Marital status**					
Single/Never married	164(48.1)	177(51.9)	341(100.0)	4.81	**0.039** #
Married/co-habiting	1(11.1)	8(88.9)	9(100.0)		
**Year of study**					
100–200	63(49.2)	65(50.8)	128(100.0)	0.997	0.607
300	48(49.0)	50(51.0)	98(100.0)		
400–600	54(43.5)	70(56.5)	124(100.0)		
**Family History of Breast cancer**					
No	153(47.5)	169(52.5)	322(100.0)	0.224	0.636
Yes	12(42.9)	16(57.1)	28(100.0)		
**Knowledge of breast cancer**					
Good	148(46.8)	168(53.2)	316(100.0)	0.123	0.725
Poor	17(50.0)	17(50.0)	34(100.0)		
**Attitude towards breast cancer** **screening**					
Negative	10(100.0)	0(0.00)	10(100.00)	11.542	**<0.001** #
Positive	155(45.6)	185(54.4)	340(100.00)		
**Practice of CBE**					
	334(95.4)	16(4.6)	350(100.0)		
**Age (years)**					
16 – 20	166(95.4)	8(4.6)	174(100.0)	0.001	0.981
21 – 35	168(95.5)	8(4.5)	176(100.0)		
**Marital status**					
Single/Never married	325(95.3)	16(4.7)	341(100.0)	0.443	1.000#
Married/co-habiting	9(100.0)	0(0.0)	9(100.0)		
**Year of training**					
1^st^ – 2^nd^	120(93.8)	8(6.2)	128(100.0)	1.422	0.491
3^rd^	95(96.9)	3(3.1)	98(100.0)		
4^th^ – 6^th^	119(96.0)	5(4.0)	124(100.0)		
**Family History of breast cancer**					
No	307(95.3)	15(4.7)	322(100.0)	0.070	1.000#
**Yes**	27(96.4)	1(3.6)	28(100.0)		
**Knowledge of breast cancer**					
Poor	301(95.3)	15(4.7)	316(100.0)	0.229	1.000#
Good	33(97.1)	1(2.9)	34(100.0)		
**Attitude towards breast cancer** **screening**					
Negative	10(100.0)	0(0.00)	10(100.0)	0.493	1.000#
Positive	324(95.3)	16(4.7)	340(100.0)		

# Fisher's exact p value

X^2^ Chi square test

## Discussion

Our study showed that female undergraduates had poor knowledge of breast cancer and screening, but majority had positive attitude towards screening for it. They had good practice of breast self-examination while practice of clinical breast examination was considerably low. Personal attributes such as year of study was significantly associated with knowledge, while marital status and attitude significantly influenced screening (breast self-examination).

The respondents in this study were mostly young and single. This is expected as the minimum entry age into Nigeria tertiary institutions is 16 years and many undergraduate females would prefer to get married after graduation from school. This is similar to the findings of a study conducted among university students in Ethiopia.^7^ The respondents in Ibadan, Southwest Nigeria were older as the study was carried out among female postgraduate students. 8 Also, respondents in Port Harcourt and Borno in Nigeria as well as Kampala in Uganda were mostly single. 9,10,11 Similarly, other studies carried out in Oyo State and Port Harcourt in Southern Nigeria reported preponderance of Christians among their respondents.^5^,^10^ Christianity is the predominant religion in Southern Nigeria hence the finding.

The high awareness of breast cancer recorded in this study which was reported by the respondents to have been from media exposure is identical to findings from another study.^12^ This is because as youths and university undergraduates, mass media platforms and internet are easily accessible to them. A study with identical objectives found that women who had tertiary education were more knowledgeable about breast cancer than those who had less education.^13^ Like in the Lagos study mentioned above, few 28(8.0%) of our respondents had family history of breast cancer. 12 There may have been some under-reporting as the figure may be higher than this because respondents are not likely to be aware of the health status of all their first-degree relatives.

The poor knowledge of risk factors of breast cancer and its symptoms recorded from this study was probably as a result of insufficient information as also reported from other studies. This is because more than a third of the respondents in this study did not know about the risk factors of breast cancer which is like observations from one study conducted in Ajman, United Arab Emirates (UAE).^14^

The poor knowledge of symptoms of breast cancer recorded in this study is similar to that obtained from an Indian study.^15^ This is expected as respondents were not well-sensitized on breast cancer and screening. They do not really have in-depth knowledge about breast cancer. However, a sharp contrast is noticed in another study done among female students at a University in Uganda, East Africa where it was discovered that majority of the respondents had good knowledge on symptoms of breast cancer.^9^ The low level of knowledge in this study may be an indication that little coverage is given to health education programs on breast cancer screening in Nigerian Universities.

Most of the respondents knew surgery and chemotherapy as treatment options. In another study also conducted in Southwest Nigeria, female secondary school teachers had better knowledge of treatment methods but as much as 22.5% of them believed that surgery is the only method of treatment for breast cancer.^16^ They probably had better knowledge due to their teaching profession and older age.

Most of the students were generally aware of the screening methods. The proportion of the students who had heard of breast self-examination was higher than clinical breast examination. Awareness of mammography was the least. This finding is like that of a study conducted among female postgraduate students at University of Ibadan.^17^ Generally, majority of the respondents had poor knowledge of breast cancer screening which is similar to what was obtained from another study carried out in Southwest Nigeria.^10^ This is due to lack of adequate education among the respondents about breast cancer screening. The small proportion 30(16.2%) of the students who practiced breast self-examination monthly irrespective of whether it was done correctly or timely, was likely to be an indication that the students lack detailed information about the four screening modalities.

Our study showed that the respondents had not received any training on the practice of breast cancer screening and that the information they claimed to have on the screening measures was inadequate, hence did not translate into regular, correct and appropriate practices of the preventive measures for early detection of breast cancer. Similarly, low practice of breast self-examination has been recorded among market women who had lower levels of education.^18^ It could be inferred that most of the students who participated in our study did not know much about the practice of screening (i.e., breast self examination and clinical breast examination).

Since media was the major source of information on breast cancer screening, it may be utilized more to ensure wider coverage. In addition to wider coverage, improvement in the depth of information through these media is important since awareness is high but knowledge is poor. In University of Ibadan, mass media was also the major source of information on screening methods.^8^ Many undergraduates understandably would have access to various mass media platforms.

The clinical breast examination rate observed in this study is very low when compared with a study conducted in Ibadan among female postgraduate students; a situation where more than 25% had undergone clinical breast examination.^17^ This is likely because of older age of the postgraduate students.

With regards to attitude to breast cancer screening, students in Behshahr, Iran and Cameroon had positive attitudes. 19,20 Majority of the respondents in Cameroon stated that they were motivated by publicity and media campaigns to perform BSE.^19^

Our study showed that students in their third year of training had significantly better knowledge of breast cancer. Better income influenced attitude positively and year of study also influenced practice of BSE among respondents. Some of these findings are comparable to those noted among women in Behshahr, Iran except that in the Iranian study, level of literacy influenced attitude.^20^

The result of this study shows no statistically significant association between knowledge of breast cancer and screening and preventive practice in women which contradicts reports from Iran.^21^ Knowledge of breast cancer and screening did not influence the practice of clinical breast examination. This was the case even among female Postgraduate students at the University of Ibadan, Nigeria who are older.^17^

Further analysis (multivariable logistic regressions) indicated that being in the third year of training predicts good knowledge of breast cancer and screening AOR (95% CI) 5.87 (2.17 – 15.86). This was not quite the case with results obtained from an Ethiopian University.^22^ In that study, fifth year of training was the reference point and though the odds of better knowledge were two times more in the third year of training, it was not a predictor AOR (95% CI) 2.661 (0.407–17.389).^22^

Also, marital status and attitude towards breast cancer screening were shown to be significantly associated with practice of BSE. However, after controlling for confounding factors, no predictors were found. In other Ethiopian institutions, there was no significant relation between socio-demographic variables and practice of BSE.7,^23^ But in a Malaysian study, being married and adherence to breast self examination were predictors of practicing breast self examination.^24^ Married women may be at a slightly better advantage due to spousal support. With regards to the practice of clinical breast examination, unlike this study, being married, good knowledge of breast cancer and social support for breast cancer screening were found to be predictors of practicing clinical breast examination in the Malaysian study.^24^ This difference can be attributed to the younger and largely unmarried constitution of the respondents in our study.

## Strengths and limitations of the study

The study was conducted among female youth which constitutes an important population in the topic of study. Respondent sampling was done following rigorous scientific methods for quality assurance.

A potential weakness that should be considered when interpreting this study is that the information was collected using a self-administered questionnaire and hence the possibility that some respondents may have given incorrect information due to their inability to correctly recall past experiences. Also, the study was cross-sectional, therefore caution should be exercised in drawing causal conclusions. The study was carried out in only one institution and therefore might not be representative of other universities of the country and youth in general, and the practice of breast self examination may be different in other sectors of the population.

## Conclusion

Overall knowledge of breast cancer and breast cancer screening was poor. Majority of the respondents had positive attitude towards breast cancer screening. Most of the respondents had good practice of BSE however, practice of CBE was considerably low. Year of study was significantly associated with knowledge while marital status and attitude influenced screening (BSE). Being in the third year of training predicts good knowledge. Health education programmes on breast cancer and screening among female undergraduates in Nigeria conducted by tertiary institutions, health care providers and relevant agencies are highly recommended, putting into consideration the year of training of the students. Health care providers should consider using each encounter with female students as an avenue to educate them and also conduct CBE. Also, breast cancer education should be embedded into educational institutions' general study programmes. Further research to better explain the poor knowledge and practice of breast cancer screening even among university undergraduates are suggested, including evaluation of existing intervention programmes.
